# The ever expanding scope of electrospray mass spectrometry—a 30 year journey

**DOI:** 10.1038/s41467-019-11747-z

**Published:** 2019-09-09

**Authors:** Matthias Mann

**Affiliations:** 1 Proteomics and Signal Transduction, Max Planck Institute of Biochemistry, Martinsried, Germany; 2 NNF Center for Protein Research, University of Copenhagen, Copenhagen, Denmark

**Keywords:** Proteomics, Mass spectrometry, Proteomic analysis, Bioanalytical chemistry

## Abstract

John Fenn’s electrospray mass spectrometry (ESMS) was awarded the chemistry Nobel Prize in 2002 and is now the basis of the entire field of MS-based proteomics. Technological progress continues unabated, enabling single cell sensitivity and clinical applications.

## Early days of ESMS

The phenomenon of electrospray, in which a liquid disperses into small droplets under the influence of an externally applied electric potential has been studied for more than a hundred years. Malcom Dole first thought to embed large molecules into a liquid being electrosprayed, but it was John Fenn at Yale University who applied his knowledge of molecular beam expansion into vacuum to successfully couple ES to MS for the first time. Supplying a high voltage to a hypodermic needle placed in front of a simple mass spectrometer, his co-worker Masamichi Yamashita electrosprayed various substances dissolved in methanol:water. To their surprise, they observed ions representing the intact form of very labile molecules such as vitamin B12 and small cyclic peptides in their ES mass spectra, which had always decomposed with other ionization methods^1^. However, research into ESMS remained an esoteric and basically unfunded activity for nearly ten years^2^. This changed only when two of his Ph.D. students—Chin Kai Meng and myself—produced the first mass spectra of intact proteins without any sign of fragmentation in 1988, a feat that had eluded the MS community for many years^3,4^. Suddenly ESMS was trust from obscurity into the limelight, especially when *Science* asked us to write this up in a general review^5^. Even from then on it was not an entirely smooth journey because in the very same year—by an improbable coincidence—matrix-assisted laser desorption/ionization (MALDI), a completely different technique for protein analysis by MS, was also introduced^6^. However, far from outcompeting ESMS in protein mass spectrometry, MALDI-MS is today mostly used in specialized applications such as imaging mass spectrometry, mainly because it is not naturally compatible with liquid separation methods.

## The mysteries of electrospray

The technological prerequisites for ESMS were clearly in place many years before it was actually developed. A reason for this ‘delay’ was probably that mass spectrometrists thought in terms of producing ions in the vacuum of the mass spectrometer itself. This was the basis of all other ionization techniques and should in principle be much more efficient because it avoids losses when transferring ions from atmosphere across a more than a million-fold pressure difference into high vacuum. As often in science, it took an outsider to get the field unstuck. In this case it was John Fenn’s intimate knowledge of the atmosphere to high vacuum transition and his intuition that one needed to provide enthalpy in the form of a gas atmosphere to gently desolvate ions from their liquid surrounding. That said, much of the progress in sensitivity of ESMS over the last decades has been in recouping as much as possible of the inevitable ion transfer loss. The signal in ES is proportional to the concentration of the analyte rather than the total amount. Therefore, reducing the flowrate drastically improves overall sensitivity. We showed that ‘nanoelectrospray’ (nanoES), which operates at flowrates of low nanoliters per minute, is capable of detecting one in a few hundred of the ions originally produced in the ES source, as opposed to one in hundreds of thousands for regular, high flow ES sources^7^. Combined with robust sample preparation techniques and database searching by peptide sequence tags, this made MS competitive with chemical techniques for protein analysis and led to the characterization of a large number of key biological molecules^8^.

Contrasting with its successful application, our theoretical understanding of the physico- and electro-chemical processes in ES has not advanced as much over the years as one might have expected. Despite intricate theories in my original Ph.D. thesis and elsewhere, it is still not entirely clear how proteins are actually ionized from the electrosprayed droplets^9,10^. Likewise, while it is well known that nanoES is more efficient, more robust towards impurities and provides a more quantitative ionization response across different biomolecules such as peptides, why this is and how we could use this knowledge to make ES more efficient at higher flow rates remains unclear.

One interesting approach to improve ES features multi-sprayers, which step down the flowrate towards that of the ideal nanoES range^11^. Even more intriguingly, modern concepts of radiofrequency containment of electrosprayed ions in an intermediate pressure range of a few millibar may finally solve the problem of ion loss at the ES to MS interface. In such a subambient ionization (SPIN) source, the effluent of a nanobore column would be sprayed directly into an electrodynamic ‘funnel’, from which ions can be transported very efficiently to subsequent stages^12^. Thus, I predict that ionization sources with close to 100% efficiency from ion generation to MS will eventually appear in the future.

This still leaves the fundamental issue—also hardly addressed for 30 years—that different molecules have different ‘flyabilities’, meaning they generate different MS signals even when present at the same concentrations. For instance, when digesting a protein into peptides, these peptides will appear in the mass spectrometer with very different signals, mainly as a consequence of different propensities to take on a charge. One possible solution could be to chemically tag all produced peptides with an easily ionizable group, in effect normalizing the response. This would not be a drastically more complex experiment as peptides are already routinely chemically modified for their relative quantitation in isobaric tagging procedures. However, downstream analysis in proteomics experiments would then be even more overwhelmed with all the peptides of highly abundant proteins, so this may not be a feasible solution for total proteome analysis at this point.

## Towards the ideal mass spectrometer

Today, mainstream proteomics instruments combine a quadrupole for mass selection, often an ion trapping device of some form, a device for fragmenting the analyte and a readout section to produce the actual mass spectra. Mass spectrometers have improved by orders of magnitude in terms of mass resolution, sensitivity and sequencing speed. Interestingly, with the notable exception of the Orbitrap analyzer^13^, this was achieved through the refinement of individual components such as the ion trap, quadrupole and the time of flight analyzers, which were all introduced more than 60 years ago. Much progress was driven by the demand to utilize the increasing ion currents produced by modern ES sources, which can now generate ions in the order of a billion per second. This flux presents a fundamental challenge for any trapping instrument because they all have a limited ion capacity and readout time. So far, there is no optimal solution to the ion storage problem, but doing without storage would waste all the ion species not selected for analysis at a given time. Arguably, the reasons for the demise of the 3D ion trap in proteomics were not only its poor mass resolution and mass accuracy but also its very limited charge capacity. More recent developments, such as the C-trap that stores ions for injection into the Orbitrap, also cannot handle more than about a million ions, fundamentally limiting the dynamic range of single mass spectra and the proportion of all ions sampled from the source. This problem is partially addressed by the BoxCar method, which effectively normalizes the ion load over the mass range, or by data independent acquisition (DIA), which only stores the fragments of a small mass range of the incoming ion species^14,15^. However, given the vast dynamic range of protein concentrations in body fluids such as plasma, it is not clear whether this will be sufficient. A more general solution could again come from confining ions in the intermediate pressure range. Storage of up to a billion ions is possible in such devices, although the interface to MS analysis has not been convincingly solved yet^16^. However, as storage of all the ions is physically possible, this may happen in the foreseeable future.

In summary, ESMS has come a very long way since the demonstration that it can ionize large molecules 30 years ago. The pace of innovation has not diminished, and arguably we are even getting close to theoretically ideal instrumental performance in several analytical dimensions. There are huge opportunities for mass spectrometry in clinical applications, such as plasma proteome profiling for precision medicine or cancer tissue proteomics^17^. Thus, there is hope that all of the investments in ESMS technology and adjacent areas will be handsomely rewarded not only in the fields it was originally developed for but also in terms of improving public health—a topic none of us had even imagined thirty years ago.
